# Mechanical and Dielectric Properties of Epoxy Resin Toughened by a Hydroxyl-Terminated Hyperbranched Polymer

**DOI:** 10.3390/polym18151874

**Published:** 2026-07-30

**Authors:** Haibin Zhou, Jun Deng, Zhicheng Xie, Zhicheng Pan, Yanjie Cui, Dong Yue, Yu Feng, Minghe Chi, Xunjun He

**Affiliations:** 1 Electric Power Research, Institute of Extra High, Voltage Transmission Company China Southern Power Grid Co., Ltd., Guangzhou 510663, China; 990413968748138@163.com (H.Z.); reacherempire@163.com (J.D.); zcxie0609@139.com (Z.X.); panzhichenghk@126.com (Z.P.);; 2 School of Electrical and Electronic Engineering, Harbin University of Science and Technology, Harbin 150080, China

**Keywords:** epoxy resin, hydroxyl-terminated hyperbranched polymer, toughening modification, mechanical properties, dielectric properties, electrical insulation

## Abstract

Epoxy resin (EP) has been extensively used in electrical insulation systems because of its favorable adhesion, chemical resistance, thermal stability, and dielectric reliability. Nevertheless, the dense three-dimensional network formed during curing generally gives EP a brittle nature, which restricts its use in insulating components that require both mechanical robustness and long-term reliability. In this work, a hydroxyl-terminated hyperbranched polymer (HBP-OH) was synthesized from itaconic acid (IA) and dipentaerythritol (DPE) through an Ax + By polycondensation route and then incorporated into an anhydride-cured epoxy system as a reactive toughening component. The influence of HBP-OH on the structure, mechanical behavior, dielectric response, and DC breakdown strength of the resulting HBP-OH/EP composites was systematically evaluated. The results demonstrate that HBP-OH effectively improves the mechanical performance of EP. At an HBP-OH loading of 9 wt%, the tensile strength increased from 15.00 MPa for pure EP to 33.27 MPa, while the elongation at break and flexural strength reached 6.5% and 88 MPa, respectively. Meanwhile, only a slight reduction in DC breakdown strength was observed at the optimal HBP-OH content. These results indicate that the proposed hyperbranched-polymer modification strategy can improve the toughness and strength of epoxy resin while retaining its dielectric and insulation performance, providing a feasible approach for developing epoxy insulating materials for high-voltage electrical equipment.

## 1. Introduction

Epoxy resin (EP) is a representative thermosetting polymer used in high-voltage insulation because it combines strong adhesion, good dimensional stability, chemical resistance, and reliable dielectric behavior. For anhydride-cured epoxy systems, the curing reaction, accelerator concentration, and molecular architecture jointly determine the final network structure and therefore affect the dielectric response and service stability of the material [1]. Recent studies have suggested that introducing charge-blocking interfaces or molecular trapping sites can improve breakdown strength and reduce dielectric loss in epoxy-based insulating materials [2,3]. However, the highly cross-linked structure of cured EP also limits chain mobility and plastic deformation, leading to brittle fracture under mechanical, thermal, or electrical stress [4]. Therefore, designing epoxy systems with improved toughness without sacrificing insulation performance remains an important issue for high-reliability electrical applications. Recent studies have summarized or demonstrated several modification routes, including nanofiller toughening, polyurethane modification, rubber/thermoplastic hybrid toughening, and self-assembled nanostructure regulation [5,6,7,8].

Filler-based strategies can regulate epoxy performance by creating interfacial regions, changing segmental relaxation behavior, and affecting charge transport pathways. Dielectric spectroscopy combined with activation-energy analysis has been used to reveal the relationship between relaxation processes and dielectric behavior in hybrid epoxy composites [9]. Molecular dynamics simulations also provide insights into how graphene-based fillers influence heat transfer and dielectric behavior by changing local chain motion and interfacial interactions [10]. In addition, boron nitride/epoxy composites prepared under high-frequency electric fields have shown improvements in electrical, thermal, and mechanical properties [11]. Beyond filler incorporation, structural design has also been used to enhance epoxy systems; for example, architectured epoxy composite lattices fabricated by 3D printing can achieve strength and toughness improvements through a combination of material design and structural optimization [12]. Nevertheless, inorganic fillers may increase viscosity and tend to agglomerate at high contents, which can create defects and local electric-field distortion that are unfavorable for breakdown performance.

Reactive molecular toughening offers another pathway because the modifier can participate in network formation and improve compatibility with the epoxy matrix. Hyperbranched epoxy resins have been shown to enhance both flame retardancy and toughness through multifunctional groups and energy-dissipation mechanisms [13]. Hydroxyl-terminated phenolphthalein polyether sulfone has also been applied to toughen epoxy resin by improving matrix compatibility and stress transfer [14]. Similarly, hydroxyl-terminated polybutadiene-based polyurethane resin can increase the toughness and strength of room-temperature-curing epoxy adhesives by introducing flexible chains and reinforcing interfacial bonding [15]. Multifunctional phosphorus-, silicon-, and bio-based modifiers further demonstrate that reactive molecular design can simultaneously regulate toughness, flame retardancy, transparency, and other functional properties of epoxy systems [16,17]. Chitosan-based cross-linking and POSS/polyoxometalate supramolecular nanocrystals confirm that interfacial interaction, dynamic bonding, and multiscale energy dissipation are effective factors for strengthening and toughening epoxy thermosets [18,19]. Organic–inorganic core–shell nanoparticles and reactive thermoplastic modifiers can also improve fracture resistance by controlling phase morphology and crack propagation behavior [20,21].

Hyperbranched polymers have also been explored in epoxy dielectric materials through several modification routes. Hyperbranched aromatic polyamide has been grafted onto boron nitride nanoplatelets to improve filler dispersion, thermal conductivity, and breakdown strength [22]. Carboxyl-terminated hyperbranched polyester has also been used to treat nanosilica and regulate interfacial bonding, charge trapping, and dielectric relaxation [23]. In these systems, the hyperbranched polymer mainly acts as an interfacial layer, and performance remains dependent on inorganic-filler loading and dispersion. Flexible-chain-capped hyperbranched polyesters have also been added directly to epoxy to improve toughness and reduce dielectric constant and loss [24,25]. End-group capping reduces polar-group density but also decreases the number of reactive terminals available for integration into the curing network. Thus, the direct use of an uncapped, hydroxyl-rich hyperbranched polyester as a reactive toughener for balancing mechanical reinforcement and high-voltage insulation remains insufficiently investigated.

For electrical insulation, toughening modification must be evaluated together with dielectric stability and breakdown reliability. Schiff-base epoxy insulating materials have been investigated to combine electrical performance with degradable molecular structures [26]. Studies on electrical treeing show that mechanical stress can accelerate degradation in epoxy resin, suggesting that mechanical damage and electrical aging are coupled in insulating polymers [27]. Corona aging, contamination, and condensation can also change the flashover behavior of epoxy insulating surfaces, highlighting the importance of surface stability during service [28]. Molecular structure regulation, such as benzene-ring design, has been used to balance thermal and electrical properties in epoxy dielectric polymers [29]. In addition, multifunctional additives have been developed to improve mechanical properties, low-dielectric behavior, and flame retardancy, indicating that integrated structure design is essential for multifunctional epoxy insulation [30].

Apart from property enhancement, sustainability and recyclability are increasingly important in the development of epoxy thermosets. Alcoholysis reconstruction has been reported as a method for epoxy recycling while maintaining or improving breakdown strength after reconstruction [31]. Closed-loop recycling of tough supramolecular epoxy thermosets based on hyperbranched topologies further indicates that a branched molecular design can contribute to both toughness and recyclability [32]. Reviews of degradable epoxy structures and dynamic cross-linking networks also show that recyclable epoxy resins can be developed without completely losing mechanical performance [33]. For insulating epoxy materials, hot-press recycling and alcohol degradation of anhydride-cured networks provide a useful understanding of network reconfiguration and property evolution [34,35]. Hygrothermal aging studies of epoxy composites for medium-frequency transformers further reveal that environmental stability is critical for practical insulation reliability [36]. Recent progress in toughening and degradation strategies confirms that molecular-level design is a promising route for simultaneously improving toughness, durability, and sustainability in epoxy thermosets [37].

Cast-resin dry-type transformer insulation must withstand high electric fields while providing mechanical support under short-circuit forces, thermal cycling, and operational stresses. This dual requirement makes it necessary to reduce epoxy brittleness without markedly impairing dielectric reliability. In this work, HBP-OH was synthesized from itaconic acid and dipentaerythritol and directly introduced into an anhydride-cured DGEBA system [38]. Unlike filler-grafted or flexible-chain-capped hyperbranched modifiers, this HBP-OH retains abundant terminal hydroxyl groups. These groups may promote compatibility and participate in the anhydride–epoxy curing process. Meanwhile, flexible ester-containing branches may provide local free volume, enable segmental rearrangement, and dissipate mechanical energy. Two competing effects are therefore anticipated. Greater free volume and segmental mobility may promote plastic deformation but can increase polarization and reduce electrical strength. Conversely, reactive network incorporation and intermolecular interactions may improve stress transfer and restrain excessive segmental motion. The novelty of this study lies in using an IA/DPE-derived reactive HBP-OH to regulate this competition and evaluating the resulting mechanical–electrical property balance for potential cast-resin dry-type transformer applications.

## 2. Materials and Methods

### 2.1. Materials

Bisphenol A epoxy resin (DGEBA) was supplied by Nantong Xingchen Synthetic Materials Co., Ltd. (Nantong, China). Methyl hexahydrophthalic anhydride (MeHHPA), used as the curing agent, was obtained from Changzhou Runxiang Chemical Co., Ltd. (Changzhou, China). The accelerator 2,4,6-tris(dimethylaminomethyl)phenol (DMP-30, purity ≥ 96%) was purchased from Shanghai Macklin Biochemical Technology Co., Ltd. (Shanghai, China). Dipentaerythritol (DPE, purity ≥ 90%), itaconic acid (IA, purity ≥ 99%), N-methylpyrrolidone (NMP), and xylenes were purchased from Beijing Inokai Technology Co., Ltd. (Beijing, China). All reagents were used as received unless otherwise stated.

### 2.2. Preparations

**Figure 1:**
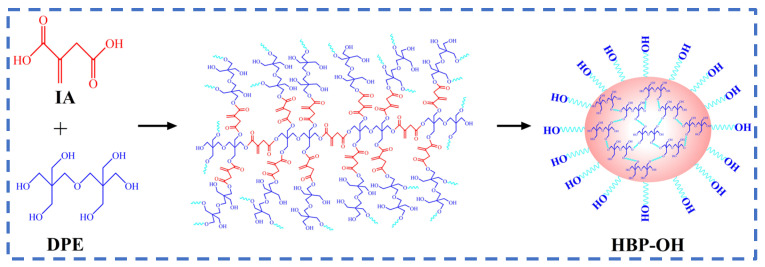
Synthesis route of the hydroxyl-terminated hyperbranched polymer (HBP-OH).

HBP-OH was synthesized through the polycondensation reaction between IA and DPE. First, 4.67 g of IA and 25 mL of NMP were transferred into a 250 mL three-necked flask equipped with a condenser, a nitrogen inlet, an addition port, and a magnetic stirrer. The nitrogen inlet was used to provide an inert reaction environment. The reaction mixture was heated to 130 °C under stirring at 1000 rpm. To favor the formation of hydroxyl-rich hyperbranched structures, 9.41 g of DPE was divided into three equal portions and added at reaction times of 0, 3, and 6 h. After the final addition, the reaction was continued for another 3 h, giving a total reaction time of approximately 9 h. The proposed synthetic route of HBP-OH is shown in Figure 1.

After the reaction, the mixture was cooled to room temperature and slowly introduced into xylenes, which served as a nonsolvent to precipitate HBP-OH. Purification consisted of a single precipitation–centrifugation step, and no additional washing or reprecipitation procedure was recorded. The supernatant was removed, and the obtained pale-yellow viscous precipitate was dried at 80 °C for 10 h. The dried pale-yellow product was subsequently ground into a powder. The precipitation and drying procedures were intended to reduce residual NMP, unreacted monomers, and soluble low-molecular-weight species.

For the preparation of HBP-OH/EP composites, DGEBA was first mixed with MeHHPA at 80 wt% relative to the mass of DGEBA. HBP-OH was then added at 0, 3, 6, 9, and 12 wt% relative to the mass of DGEBA, followed by DMP-30 at 1 wt% relative to the mass of DGEBA. The mixture was mechanically stirred at room temperature for 120 min and then degassed under vacuum for 120 min. Specimens for mechanical testing were poured into preheated molds and cured in an oven at 100 °C for 2 h, followed by 120 °C for 2 h. Specimens for electrical testing were prepared as films with a thickness of approximately 200 μm using a flat vulcanizing press under the same curing-temperature program. The same batch of HBP-OH was used for all composite formulations.

### 2.3. Characterizations

The chemical structures of IA, DPE, HBP-OH, pure EP, and the HBP-OH/EP composites were analyzed by Fourier transform infrared spectroscopy using the KBr-pellet method over the wavenumber range of 4000–400 cm^−1^. X-ray diffraction measurements were performed using an Empyrean X-ray diffractometer (PANalytical, Almelo, The Netherlands) with Cu Kα radiation (*λ* = 0.154 nm) at 40 kV and 40 mA at room temperature. Differential scanning calorimetry was conducted under a nitrogen atmosphere at a heating rate of 10 °C/min. HBP-OH samples of approximately 5–10 mg were scanned from 25 to 250 °C, whereas the cured epoxy samples were scanned from 25 to 200 °C. The molecular weight of HBP-OH was measured using a PL-GPC200 gel permeation chromatograph with DMF as the mobile phase. The ^1^H NMR spectrum of HBP-OH was recorded using an Avance Neo 400 nuclear magnetic resonance spectrometer (Bruker, Bremen, Germany) with deuterated pyridine as the solvent.

**Figure 2:**
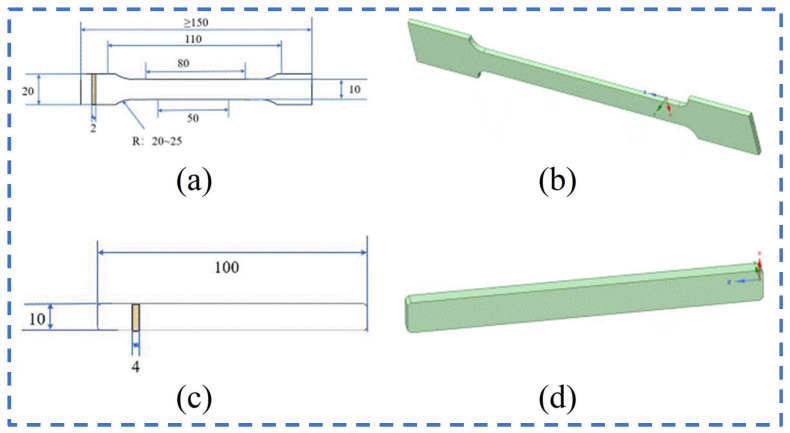
(**a**) Planar dimensions and (**b**) 3D model of the dumbbell-shaped tensile specimen; (**c**) planar dimensions and (**d**) 3D model of the rectangular flexural specimen.

The tensile-fracture surfaces of pure EP and the HBP-OH/EP composites were mounted on conductive adhesive, sputter-coated with gold, and observed using an SU8020 field-emission scanning electron microscope (Hitachi, Tokyo, Japan). All cured specimens were tested in the as-cured state without any additional conditioning or pre-treatment. Tensile tests were performed at room temperature using an E44.104 universal testing machine at a crosshead speed of 5 mm/min according to GB/T 2567-2008. The dumbbell-shaped specimens had an overall length of at least 150 mm, a gauge length of 50 mm, a narrow-section width of 10 mm, and a thickness of 2 mm, as shown in Figure 2a,b. Flexural properties were measured by three-point bending at room temperature according to GB/T 9341-2008 using specimens with dimensions of 100 mm × 10 mm × 4 mm and a loading rate of 1 mm/min, as shown in Figure 2c,d.

Dielectric properties were measured using an Alpha-A broadband dielectric spectrometer (Novocontrol, Montabaur, Germany) over a frequency range of 10^1^–10^6^ Hz. The electrical specimens had a thickness of approximately 200 μm. DC breakdown strength was tested at room temperature according to IEC 60243-2 using a YDZ-5 precision DC power supply and a YDK-type control console. The specimens were immersed in silicone oil, and the voltage was increased at a rate of 1 kV/s. A cylindrical electrode was used as the high-voltage electrode, and a plate electrode was used as the grounded electrode. Twenty breakdown points were tested for each formulation, and the results were analyzed using a two-parameter Weibull distribution.

## 3. Results and Discussion

### 3.1. Structural Characterization of Dipentaerythritol, Itaconic Acid, and Hydroxyl-Terminated Hyperbranched Polymers

**Figure 3:**
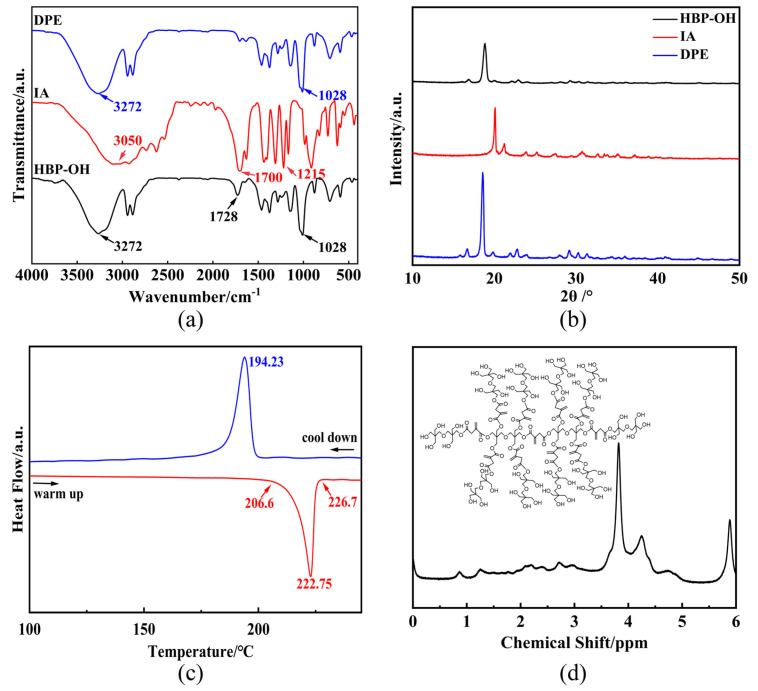
(**a**) FTIR spectra and (**b**) XRD patterns of DPE, IA, and HBP-OH; (**c**) DSC heating and cooling curves of HBP-OH; (**d**) ^1^H NMR spectrum and the corresponding chemical structure of HBP-OH.

The FTIR spectra of DPE, IA, and HBP-OH are presented in Figure 3a. For DPE, the absorption at 1028 cm^−1^ is associated with the stretching vibration of the ether bond (-C-O-C-), and the broad band at 3272 cm^−1^ originates from associated hydroxyl groups. For IA, the absorption at 1700 cm^−1^ corresponds to the carbonyl group in carboxyl groups, the peak at 1215 cm^−1^ is related to C-O vibration, and the broad band near 3050 cm^−1^ is assigned to O-H vibration. After the reaction, HBP-OH shows a new ester carbonyl absorption at 1728 cm^−1^. Meanwhile, the characteristic carboxyl signals of IA are markedly weakened or disappear, and the hydroxyl absorption becomes more distinct. These spectral changes indicate that ester linkages were formed through the reaction between IA and DPE, confirming the successful preparation of hydroxyl-terminated HBP-OH.

Figure 3b shows the XRD patterns of DPE, IA, and HBP-OH. DPE and IA exhibit strong diffraction peaks at 2*θ* = 18.57° and 2*θ* = 20.11°, respectively, indicating their crystalline characteristics. In contrast, HBP-OH displays a new diffraction peak at 2*θ* = 18.87° and a pattern different from those of the raw materials, suggesting the formation of a new polymeric structure after polycondensation. The DSC curves are shown in Figure 3c. HBP-OH shows a melting region rather than a sharp melting point, which is consistent with the molecular-weight distribution of hyperbranched polymers. The onset melting temperature is 206.6 °C, and complete melting occurs at 226.7 °C. The main endothermic peak appears at 222.75 °C, indicating the relatively stable thermal transition behavior of the synthesized HBP-OH. The disappearance or substantial weakening of the characteristic carboxyl absorptions of IA, together with the formation of the ester-carbonyl band, supports the conversion of IA and DPE into HBP-OH. In addition, no separate melting peaks corresponding to crystalline IA or DPE were observed in the DSC curve, and the XRD pattern of HBP-OH differed from those of both monomers. These results indicate that no large amount of crystalline unreacted monomer remained in the recovered product.

GPC was used to determine the molecular weight and dispersity of HBP-OH, and the results are listed in Table 1. The polydispersity index (PDI) was calculated according to Equation (1):

(1) $$PDI=\frac{Mw}{Mn}$$

where *M*_w_ and *M*_n_ represent the weight-average and number-average molecular weights, respectively. The calculated *PDI* of HBP-OH is 2.09, showing a relatively broad molecular weight distribution, which is a typical feature of hyperbranched polymers.

The ^1^H NMR spectrum of HBP-OH is shown in Figure 3d, and the corresponding proton assignments are summarized in Table 2. The signal at 2.21 ppm is attributed to methylene protons adjacent to the unsaturated double bond, while the resonance at 3.81 ppm is assigned to methylene protons near the carbonyl group. The signal at 4.24 ppm corresponds to methylene protons connected to ester oxygen, further supporting the formation of ester linkages. The peak at 5.89 ppm is associated with protons on carbon–carbon double bonds. These results are consistent with the expected structure of HBP-OH. Based on the IA/DPE feed ratio and the GPC-derived *M*_n_, and assuming that esterification was the predominant reaction, an average HBP-OH molecule was roughly estimated to contain approximately 6.57 IA units, 6.77 DPE units, 12.35 ester linkages, and 28 residual hydroxyl groups.

### 3.2. Microscopic Morphology and Structure Characterization of Epoxy Resin and Its Composites

**Figure 4:**
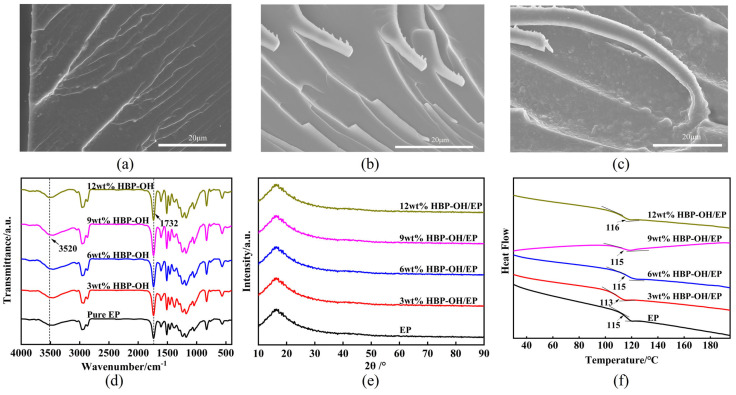
SEM images of the tensile fracture surfaces for (**a**) pure EP, (**b**) 6 wt% HBP-OH/EP, and (**c**) 9 wt% HBP-OH/EP; (**d**) FTIR spectra, (**e**) XRD patterns, and (**f**) DSC curves of the pure EP and HBP-OH/EP composites with varying HBP-OH contents.

The tensile fracture morphologies of pure EP and HBP-OH/EP composites are shown in Figure 4a–c. Pure EP exhibits a relatively flat fracture surface with river-like patterns, indicating limited plastic deformation and typical brittle failure. After introducing HBP-OH, the fracture surface becomes rougher and more complex. For the 6 wt% HBP-OH/EP composite, layered and step-like features can be observed, suggesting that crack propagation was deflected and more fracture energy was consumed. When the HBP-OH content increases to 9 wt%, more pronounced fibrillar and ductile deformation features appear on the fracture surface. This indicates that an appropriate amount of HBP-OH can promote plastic deformation and improve energy dissipation during fracture.

Figure 4d presents the FTIR spectra of pure EP and HBP-OH/EP composites. The broad absorption near 3520 cm^−1^ is assigned to hydroxyl groups, and the peaks at 1612, 1514, and 1452 cm^−1^ are related to benzene-ring vibrations. The peak at 1732 cm^−1^ corresponds to ester carbonyl groups. With increasing HBP-OH content, ester carbonyl absorption becomes stronger, which can be attributed to the ester groups introduced by HBP-OH and those generated during anhydride curing. These results suggest that HBP-OH was incorporated into the epoxy system and had good compatibility with the cured network.

The XRD patterns in Figure 4e show a broad amorphous halo around 2*θ* = 16.5° for pure EP and all composites, which is characteristic of an amorphous cross-linked epoxy network. No obvious crystalline diffraction peak from HBP-OH is detected in the cured composites, implying that HBP-OH did not form large crystalline aggregates after curing. The similar diffraction profiles of the composites and pure EP indicate that HBP-OH was well-distributed in the epoxy matrix.

The DSC curves of the cured samples are shown in Figure 4f. Each composite exhibits a single glass transition temperature without a separate transition peak from free HBP-OH, indicating good compatibility between HBP-OH and EP. The *T*_g_ values remain around 115 °C, with only slight variation as the HBP-OH content changes. This behavior may be interpreted as the result of competing molecular effects. On the one hand, the flexible ester-containing segments of HBP-OH may increase local conformational freedom and segmental mobility, which would generally tend to decrease *T*_g_. On the other hand, the abundant residual hydroxyl groups of HBP-OH may participate in the epoxy–anhydride curing reactions, promoting the covalent incorporation of HBP-OH into the cured network and consequently restricting segmental motion. Similar covalent incorporation of hydroxyl-functional hyperbranched polymers has been reported in DGEBA/anhydride thermosets, in which the addition of the hyperbranched modifier maintained or even increased *T*_g_ [22,39]. In addition, the residual hydroxyl groups may form intra- and intermolecular hydrogen-bonding interactions with polar groups in the cured network. Molecular simulation studies of hydroxyl-terminated hyperbranched polyesters have shown that such hydrogen-bonding networks can restrict local group reorientation and molecular mobility [40]. Therefore, the mobility-enhancing effect of the flexible ester-containing segments may be partially counterbalanced by the motion-restricting effects associated with possible covalent network incorporation and hydrogen-bonding interactions, resulting in only limited variation in *T*_g_.

### 3.3. Mechanical Properties of Epoxy Resin and Its Composites

**Figure 5:**
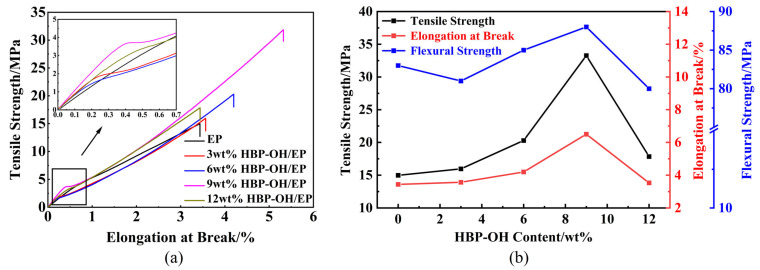
(**a**) Tensile stress–strain curves of the pure EP and HBP-OH/EP composites; (**b**) variations in tensile strength, elongation at break, and flexural strength as a function of HBP-OH content.

The tensile stress–strain curves of pure EP and HBP-OH/EP composites are shown in Figure 5a. Pure EP fails after a nearly linear elastic deformation, reflecting its brittle nature. After HBP-OH is introduced, the curves show more obvious yielding and plastic deformation before fracture. This change indicates that HBP-OH improves the deformation capacity of the epoxy matrix. The macroscopic tensile behavior is consistent with the rougher fracture surfaces and fibrillar structures observed by SEM. The hydroxyl-rich hyperbranched structure can introduce ester linkages and hydrogen-bonding interactions in the cured network, thereby increasing energy dissipation during stretching.

The mechanical-property variation with HBP-OH content is summarized in Figure 5b. Tensile strength and elongation at break first increase and then decrease as the HBP-OH content increases. The best overall tensile performance is obtained at 9 wt% HBP-OH, where the tensile strength and elongation at break reach 33.27 MPa and 6.5%, respectively. The flexural strength also reaches a maximum of 88 MPa at the same loading. At suitable contents, HBP-OH can serve as a flexible and reactive structural unit that improves stress transfer and consumes fracture energy. When the HBP-OH content is further increased to 12 wt%, the balance between the flexibility introduced by HBP-OH and the integrity of the cured epoxy network may be disturbed. The increased proportion of flexible ester-containing segments and residual hydroxyl groups may enhance local molecular mobility and alter the local curing environment, thereby reducing the efficiency of stress transfer through the network. Consequently, the mechanical properties decrease after the optimum HBP-OH content is exceeded.

**Figure 6:**
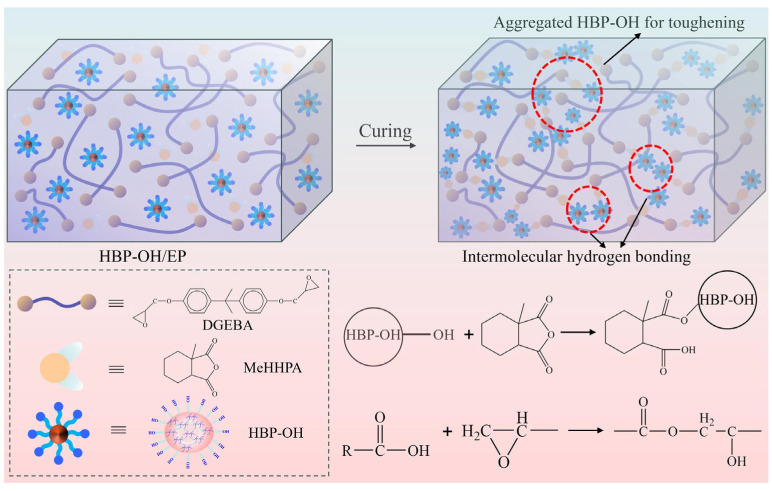
Schematic diagram of the curing process, chemical cross-linking network formation, and the toughening mechanism of the HBP-OH/EP composites.

Figure 6 illustrates the proposed network formation and toughening mechanism of HBP-OH/EP composites. During the curing process, the terminal hydroxyl groups of HBP-OH can react with anhydride groups from MeHHPA to form ester linkages and carboxyl groups. The generated carboxyl groups may further participate in epoxy ring-opening reactions, enabling HBP-OH to become part of the EP/MeHHPA network. In addition, residual hydroxyl groups can form hydrogen-bonding interactions with surrounding polar groups in the matrix.

The multifunctional DPE-derived units provide potential branching junctions, whereas the relatively short IA-derived ester-containing segments may provide local conformational flexibility. Meanwhile, the multiple residual hydroxyl groups may improve compatibility and stress transfer through possible curing reactions and intermolecular hydrogen-bonding interactions. These structural characteristics may jointly promote local plastic deformation, hinder crack propagation, and increase fracture-energy dissipation.

### 3.4. Electrical Properties of Epoxy Resin and Its Composites

**Figure 7:**
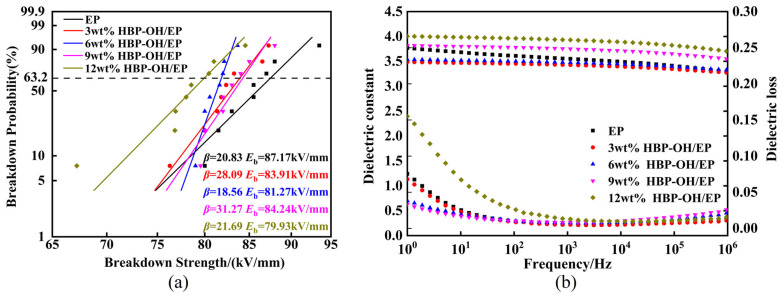
(**a**) Weibull distribution of the DC breakdown strength; (**b**) frequency dependence of the dielectric constant and dielectric loss tangent for the pure EP and HBP-OH/EP composites.

The DC breakdown strength of the composites was evaluated using a two-parameter Weibull distribution, as shown in Figure 7a. Pure EP exhibits a characteristic breakdown strength (*E*_b_) of 87.17 kV/mm. After HBP-OH is incorporated, *E*_b_ shows a slight downward tendency with increasing HBP-OH content. This behavior may be associated with the polar hydroxyl groups introduced by HBP-OH, which can enhance interfacial polarization and local charge accumulation under a DC electric field. At 12 wt% HBP-OH, *E*_b_ decreases to 79.93 kV/mm, corresponding to an 8% reduction compared with pure EP. Excessive HBP-OH may introduce more polar groups and local structural heterogeneity, thereby increasing dielectric loss and local electric-field distortion. However, the 9 wt% HBP-OH/EP composite maintains a relatively high breakdown strength while exhibiting the best mechanical performance, indicating a reasonable balance between toughening and insulation reliability.

Broadband dielectric spectroscopy was used to further examine the dielectric behavior of the samples over 1–10^6^ Hz, as shown in Figure 7b. The dielectric constant of each sample remains relatively stable at low frequencies and decreases gradually with increasing frequency. This frequency-dependent behavior is mainly caused by the delayed response of dipoles under rapidly alternating electric fields. Pure EP shows a dielectric constant of approximately 3.6. With increasing HBP-OH content, the dielectric constant first decreases slightly and then increases. At low HBP-OH contents, the consumption of hydroxyl groups during esterification and the restriction of chain mobility can suppress dipole orientation. At higher contents, remaining polar groups become more influential and lead to an increase in permittivity. The dielectric loss remains low for all samples. Moderate HBP-OH addition restricts molecular motion and helps reduce polarization loss, whereas excessive HBP-OH may increase polarization and conduction losses because of the higher concentration of polar groups and possible structural defects.

### 3.5. Comparison with Representative Epoxy-Modification Strategies

Different epoxy-modification strategies improve mechanical and electrical properties through different mechanisms. Nanoparticle-based approaches mainly rely on filler dispersion, interfacial interactions, and charge-trapping or charge-blocking effects, whereas liquid-rubber modifiers enhance toughness through rubber-particle deformation, cavitation, and matrix shear yielding. Bio-based modifiers may introduce reactive functional groups or secondary energy-dissipating phases, while reactive molecular modifiers, including hyperbranched polymers, may combine local energy dissipation with chemical or intermolecular integration into the curing network. To place the present results in the context of modern epoxy-modification strategies, representative nanoparticle-, liquid-rubber-, bio-based-, hybrid-, and reactive-modifier systems reported in the literature are compared in Table 3.

As shown in Table 3, the different modification strategies exhibit distinct property trade-offs. Nanoparticle-based systems may improve mechanical and thermal properties, but their effectiveness is closely related to filler surface functionalization, dispersion quality, and interfacial adhesion. For example, hexamethylenediamine-functionalized graphene oxide produced substantial improvements in the tensile, flexural, and impact properties of an epoxy composite at a low loading, although its electrical properties were not investigated [41]. Bio-based modifiers can also provide effective energy-dissipation mechanisms. The addition of 3 wt% acacia honey increased the absorbed energy during the first impact to approximately 2.5 times that of the unmodified epoxy and produced a high reported *T*_g_, but no dielectric or breakdown-strength data were provided [42]. Liquid rubbers generally provide effective toughening, although reductions in strength, stiffness, or thermal properties may occur depending on their type and content. In comparison, the present HBP-OH system provides substantial increases in tensile strength and elongation at break while retaining a relatively high short-time DC breakdown strength under the investigated formulation and testing conditions.

## 4. Conclusions

A hydroxyl-terminated hyperbranched polymer (HBP-OH) was synthesized from itaconic acid and dipentaerythritol and used as a reactive toughening modifier for an anhydride-cured epoxy resin system. The effects of HBP-OH content on the structure, morphology, mechanical properties, dielectric behavior, and DC breakdown strength of the HBP-OH/EP composites were investigated. The main conclusions are as follows:

1. FTIR, XRD, DSC, GPC, and ^1^H NMR analyses supported the successful synthesis of HBP-OH. The synthesized product contained ester linkages and abundant residual hydroxyl groups and exhibited a relatively broad molecular-weight distribution, consistent with the general structural characteristics of hyperbranched polymers. Based on the monomer feed ratio and the GPC-derived *M*_n_, the average molecular composition was approximately estimated;
2. HBP-OH showed relatively good compatibility with the cured epoxy matrix. FTIR, XRD, DSC, and SEM results revealed no obvious crystalline aggregation or macroscopic phase separation within the investigated specimens. The single glass-transition region was also consistent with good compatibility between HBP-OH and EP. The limited variation in *T*_g_ may result from a balance between the mobility-enhancing effect of the flexible ester-containing segments and the motion-restricting effects associated with possible network incorporation and hydrogen-bonding interactions;
3. The mechanical properties exhibited a clear dependence on HBP-OH loading, with 9 wt% providing the most favorable performance within the investigated range. At this content, the tensile strength increased from 15.00 MPa for pure EP to 33.27 MPa, while the elongation at break and flexural strength reached 6.5% and 88 MPa, respectively. The improvement may be associated with the flexible and energy-dissipating hyperbranched structure, possible curing reactions and intermolecular interactions involving the residual hydroxyl groups, and the resulting enhancement in deformation and stress-transfer capability. At 12 wt%, the mechanical properties decreased, indicating that increasing the modifier content beyond the optimum level does not continuously improve performance;
4. HBP-OH modification produced a measurable trade-off between mechanical enhancement and electrical insulation performance. At an HBP-OH content of 9 wt%, the characteristic DC breakdown strength decreased from 87.17 kV/mm for pure EP to 84.24 kV/mm, corresponding to a reduction of 3.4% and a retention of 96.6% under the present short-time laboratory test conditions. The dielectric loss remained relatively low at appropriate HBP-OH contents, whereas excessive addition increased the dielectric response and further reduced the breakdown strength;
5. The present results are limited to short-term tests on laboratory-scale specimens, and several issues require further investigation. Future work should evaluate electrical and thermal aging, partial-discharge inception and endurance, thermal conductivity, moisture and thermal-cycling resistance, and long-term dielectric reliability. Systematic comparisons with nanoparticles and liquid rubbers under identical formulation and testing conditions, preparation of HBP-OH variants with controlled molecular architectures, determination of residual solvent and low-molecular-weight species, quantitative measurement of crosslinking density, and high-resolution morphological analysis at high HBP-OH contents are also required. In addition, viscosity, mixing and degassing behavior, casting quality, void control, batch-to-batch reproducibility, and manufacturing scalability should be assessed before the material can be considered for practical high-voltage insulation applications.

Overall, HBP-OH provides a feasible molecular-modification route for regulating the mechanical–electrical property balance of anhydride-cured epoxy resin. Within the investigated range, 9 wt% HBP-OH produced the most favorable balance between mechanical enhancement and short-time DC breakdown-strength retention. These results provide a preliminary material-level basis for further development of toughened epoxy insulation systems, while long-term reliability and practical manufacturability remain to be established.

## Figures and Tables

**Table 1:** The molecular weight and polydispersity PDI of HBP-OH measured by GPC.

Molecular Weight	Value
Weight-average molecular weight *M*_w_ (g/mol)	4932
Number-average molecular weight *M*_n_ (g/mol)	2355
*PDI*	2.09

**Table 2:** Corresponding relationship between chemical structures and chemical shifts in HBP-OH.

Chemical Structure	Chemical Shift (ppm)
C=C-CH_2_-R	2.21
CH_2_-C(=O)	3.81
RCOO-CH_2_-R	4.24
R_2_C=CH-R	5.89

**Table 3:** Qualitative comparison with representative epoxy-toughening strategies reported in the literature.

Modifier/System	Loading	Reported Mechanical Outcome	Thermal/Electrical Outcome	Source
HBP-OH/DGEBA-MeHHPA	9 wt%	Tensile strength: 33.27 MPa; elongation: 6.5%; flexural strength: 88 MPa	*T*_g_: 115 °C; *E*_b_: 84.24 kV/mm; tan*δ*: 0.00869 at approximately 1.13 kHz	This work
CTBN+PEK-C/DGEBA-DDS	5 + 15 wt%	Improved impact resistance; flexural strength: −9%; modulus: −7%	No significant thermal-stability reduction; electrical properties not reported	[7]
GO-HMDA/epoxy formulation	0.1 wt%	Flexural strength: +48%; flexural modulus: +102%; impact strength: +122%; tensile strength: +82%; tensile modulus: +47%	Improved curing behavior and thermal stability; electrical properties not reported	[41]
Acacia honey/epoxy	3 wt%	First-impact absorbed energy was approximately 2.5 times that of unmodified epoxy; the specimen withstood repeated impacts	*T*_g_: 228 °C; electrical properties not reported	[42]
HBP-COOH/DGEBA-MeHHPA	6 wt%	Tensile strength: 38.79 MPa; increase of 158.6% relative to pure EP	No obvious decrease in electrical strength; dielectric loss of the same order	[24]

## Data Availability

The original contributions presented in this study are included in the article. Further inquiries can be directed to the corresponding authors.
